# TNTdetect.AI: A Deep Learning Model for Automated Detection and Counting of Tunneling Nanotubes in Microscopy Images

**DOI:** 10.3390/cancers14194958

**Published:** 2022-10-10

**Authors:** Yasin Ceran, Hamza Ergüder, Katherine Ladner, Sophie Korenfeld, Karina Deniz, Sanyukta Padmanabhan, Phillip Wong, Murat Baday, Thomas Pengo, Emil Lou, Chirag B. Patel

**Affiliations:** 1 School of Information Systems and Technology, San José State University, San José, CA 95192, USA; 2 Department of Management Information Systems, Korea Advanced Institute of Science and Technology, Daejeon 34141, Korea; 3 Department of Electronics and Communication Engineering, Yildiz Technical University, 34349 Istanbul, Turkey; 4 Department of Medicine Division of Hematology, Oncology and Transplantation, University of Minnesota Medical School, Minneapolis, MN 55455, USA; 5 Department of Neurology and Neurological Sciences, Stanford University School of Medicine, Stanford, CA 94305, USA; 6 Precision Health and Integrated Diagnostics Center, Stanford University School of Medicine, Stanford, CA 94305, USA; 7 Informatics Institute, University of Minnesota, Minneapolis, MN 55455, USA; 8 Masonic Cancer Center, Minneapolis, MN 55455, USA; 9 Department of Neuro-Oncology, MD Anderson Cancer Center, The University of Texas System, Houston, TX 77030, USA; 10 Neuroscience Graduate Program, MD Anderson UTHealth Graduate School of Biomedical Sciences, Houston, TX 77030, USA; 11 Cancer Biology Graduate Program, MD Anderson UTHealth Graduate School of Biomedical Sciences, Houston, TX 77030, USA

**Keywords:** artificial intelligence, automated cell counting, biomarker, cancer, cells, deep learning, machine learning, microscopy, TNT, tunneling nanotubes

## Abstract

**Simple Summary:**

Microscopy is central to many areas of biomedical science research, including cancer research, and is critical for understanding basic pathophysiology, mechanisms of action, and treatment response. However, analysis of the numerous images generated from microscopy readouts is usually performed manually, a process that is tedious and time-consuming. Moreover, manual analysis of microscopy images may limit both accuracy and reproducibility. Here, we used an artificial intelligence approach to analyze tunnelling nanotubes (TNTs), a feature of cancer cells that may contribute to their aggressiveness, but which are hard to identify and count. Our approach labeled and detected TNTs and cancer cells from microscopy images and generated TNT-to-cell ratios comparable to those of human experts. Continued refinement of this process will provide a new approach to the analysis of TNTs. Additionally, this approach has the potential to enhance drug screens intended to assess therapeutic efficacy of experimental agents and to reproducibly assess TNTs as a potential biomarker of response to cancer therapy.

**Abstract:**

Background: Tunneling nanotubes (TNTs) are cellular structures connecting cell membranes and mediating intercellular communication. TNTs are manually identified and counted by a trained investigator; however, this process is time-intensive. We therefore sought to develop an automated approach for quantitative analysis of TNTs. Methods: We used a convolutional neural network (U-Net) deep learning model to segment phase contrast microscopy images of both cancer and non-cancer cells. Our method was composed of preprocessing and model development. We developed a new preprocessing method to label TNTs on a pixel-wise basis. Two sequential models were employed to detect TNTs. First, we identified the regions of images with TNTs by implementing a classification algorithm. Second, we fed parts of the image classified as TNT-containing into a modified U-Net model to estimate TNTs on a pixel-wise basis. Results: The algorithm detected 49.9% of human expert-identified TNTs, counted TNTs, and calculated the number of TNTs per cell, or TNT-to-cell ratio (TCR); it detected TNTs that were not originally detected by the experts. The model had 0.41 precision, 0.26 recall, and 0.32 f-1 score on a test dataset. The predicted and true TCRs were not significantly different across the training and test datasets (*p* = 0.78). Conclusions: Our automated approach labeled and detected TNTs and cells imaged in culture, resulting in comparable TCRs to those determined by human experts. Future studies will aim to improve on the accuracy, precision, and recall of the algorithm.

## 1. Introduction

Microscopy is central to many areas of biomedical science research, including cancer. Microscopy allows researchers to understand basic pathophysiology, mechanisms of action, and also treatment response. However, analysis of the numerous images generated from microscopy readouts is usually performed manually, a process that is tedious and time-consuming. Manual analysis is also a process that may limit both accuracy and reproducibility. Machine learning (ML) and artificial intelligence (AI) approaches are emerging as a means to efficiently analyze large imaging datasets and thereby accelerate the capacity for data interpretation [1,2,3,4,5,6,7,8]. With the advent of ML/AI approaches, novel morphological features of cells that were previously not detectable, analyzable, or quantifiable in microscopy images can now be assessed for utility as emerging imaging-based biomarkers [9,10,11,12,13,14,15,16,17,18].

The field of intercellular communication has gained significant traction and interest over the past decade, catalyzed by characterization and improvement in methods of identification of extracellular vesicles and other modes of cell–cell signaling [19,20,21,22,23,24,25]. The niche of contact-dependent cell signaling mechanisms represents an emerging aspect of this field, led by (a) advances in understanding the role of tunneling nanotubes (TNTs) and their role in normal and pathologic physiology across health and disease and (b) discoveries related to tumor microtubes in glioblastoma and other cancer types as well [26,27,28,29,30,31,32,33,34,35,36,37,38,39,40,41,42,43]. The current study focuses on TNTs, which are long membranous F-actin based cell protrusions that connect cells at short and long distances and which are capable of acting as conduits for direct (often bi-directional) signaling between connected cells [44,45,46,47]. TNTs were first identified in the PC12 cell line (rat pheochromocytoma) and the term coined in 2004 by Rustom et al. [44]. Since then, this unique form of cellular protrusion has been identified in many cell types, including but not limited to immune cells, cancer cells, and neuronal cells [35,45,46,48,49,50,51,52,53]. While TNTs are ubiquitous across many cell types, we and others have shown that they are upregulated in invasive forms of cancer [45,53,54]. There is no current validated method to differentiate between TNTs from cancer as compared to non-cancer derived cells; however, the description of a longer and wider form of cell protrusion shown in an orthotopic model of malignant gliomas termed ‘tumor microtubes’ has shed light on the possible differences of this class of protrusions amongst malignant cell populations [30,36,43,55].

The function, ultrastructural characteristics, and mechanisms of TNTs are all under active investigation by many investigators [22,43,56,57,58,59,60,61]. Nonetheless, a distinct, specific, and reproducibly testable structural biomarker of TNTs has yet to be identified. This lack of a distinct biomarker has presented a challenge to this emerging field of cell biology. Thus, identification of TNTs has relied on visual identification of structural characteristics, including connections between two or more cells and the non-adherent nature of their protrusion ‘bridges’ when cells are cultured in vitro [29,54,59,60,62,63,64,65,66,67]; this latter feature helps to distinguish TNTs from other actin-based protrusions that adhere to the substratum in in vitro tissue culture and are more often associated with cell motility rather than cell–cell communication [67,68,69]. Manual visual identification of TNTs is a tedious and arduous process that also introduces the potential for lack of reproducibility. A more optimal approach to maximize reproducibility across the field would be validation and application of artificial intelligence-based approaches that could identify TNTs with high specificity and sensitivity, with excellent ability to also distinguish TNTs accurately from other forms of membrane-based extracellular extensions. A precise quantitative analysis of TNTs will help to gain statistical information to monitor the progression of various diseases. In this study we sought to construct an algorithm that accomplishes this by adopting the well-known U-Net deep learning model to segment images and detect TNTs [6].

## 2. Materials and Methods

### 2.1. Cell Lines

We used the human MSTO-211H (malignant pleural mesothelioma of biphasic histology) cell line, which was purchased from American Type Culture Collection in 2019 (ATCC, Rockville, MD, USA). Hereafter, “MSTO” will be used to refer to this cell line. The cells were grown in RPMI-1640. The culture media was supplemented with 10% fetal bovine serum (FBS), 1% penicillin-streptomycin, 1× GlutaMAX (all from Gibco Life Technologies, Gaithersburg, MD, USA), and 0.1% Normocin^TM^ anti-mycoplasma reagent (Invivogen, San Diego, CA, USA). Cells were maintained in a humidified incubator at 37 °C, with 5% carbon dioxide. We chose to plate the cells on regular tissue culture-treated plastic so that the AI training would have to overcome the inherent scratches present on plastic dishes.

### 2.2. Microscopy Imaging

Images were taken when the cells were 30–40% confluent, and individual TNTs and cells could easily be distinguished. Phase contrast images were acquired on a Zeiss AxioObserver M1 Microscope using a 20× PlanApo-Chromat objective with a numerical aperture of 0.8. A 5 × 5 set of tiled images were taken using a Zeiss Axio Cam MR camera with a pixel size of 6.7 × 6.7 µm resulting in a spatial resolution (dx = dy) at 20× of 0.335 µm/pixel. Tiled images were stitched into one image with Zen2 Blue software (Carl Zeiss Microscopy, White Plains, NY, USA).

### 2.3. Manual Identification of TNTs

TNTs were identified as previously described by our group and others [29,44,54,70]. Identification is based on three parameters: (i) lack of adherence to the substratum of tissue culture plates, including visualization of TNTs passing over adherent cells; (ii) TNTs connecting two cells or extending from one cell if the width of the extension is estimated to be <1000 nm; and (iii) a narrow base at the site of extrusion from the plasma membrane. Fiji [71] was used for creating the training images. TNTs were traced manually using the line tool and the set of annotations converted to a mask.

### 2.4. Initial Verification of TNTs Using Current Standard Methodology: Visual Identification

TNTs seen in phase contrast images appear to be elongated structures no thicker than 1 µm and ranging in length from 10 μm to over 100 µm. TNTs connect at least two cells as a straight line, occasionally making angles but not usually sinusoidal or wave-like when cells are cultured in vitro. TNTs can be comparably thinner than the cell walls in the images and occasionally become invisible in the image background. They tend to have a fairly uniform thickness from end-end, although portions along the tubes may bulge due to size of larger cargo trafficking internally; the term ‘gondola’ has been applied to describe this phenomenon in some previously published studies in the field [46,72]. TNTs often protrude from the membrane interface with a characteristically narrow or minimally cone-shaped base, in contrast to other thicker forms of cell-based podia protrusions [67]. In comparison to other cellular protrusions, TNTs uniquely have a 3-dimensional suspended nature in the substratum in vitro; these suspended TNTs can cross over other adherent cells. Although the basic TNT characteristics are familiar to researchers focused in the field of TNT cell biology, these features are not readily identifiable in previously utilized general machine learning algorithms.

### 2.5. Human Expert Review of Stitched MSTO Images and Identification of TNTs

Four human experts independently reviewed the images to detect structures meeting criteria as TNTs. The role of the human experts was to identify the presence (i.e., yes or no) of TNTs that connected two cells, rather than trying to label the TNTs on a pixel-by-pixel basis (this was left to the machine learning algorithm). After independent review, structures identified by three or four of the experts were classified by consensus as actual TNTs for analysis purposes; structures identified by two of the four experts were reviewed together by all experts and a consensus decision was made whether to classify them as actual TNTs or not; structures identified by one of the experts were not classified as actual TNTs. Next, we combined the knowledge from the human experts (the structures classified by consensus as actual TNTs) with the computational abilities of the deep learning model. We used an automated method to label TNTs on a pixel-by-pixel basis. This method was guided by the initial human-based labeling of the TNTs. Further details are provided in the Supplementary Materials.

## 3. Results

Supplementary Table S1 summarizes the results of inter-rater agreement for TNT identification among the four human experts using the Cohen’s kappa statistic. This reflects the pseudo-objective nature of TNT identification by human experts, and therefore the need for a deep learning-based algorithm to perform and quantitate TNT detection in a more reproducible manner.

### 3.1. General Approach to the Automated Detection of TNTs

**Figure 1:**
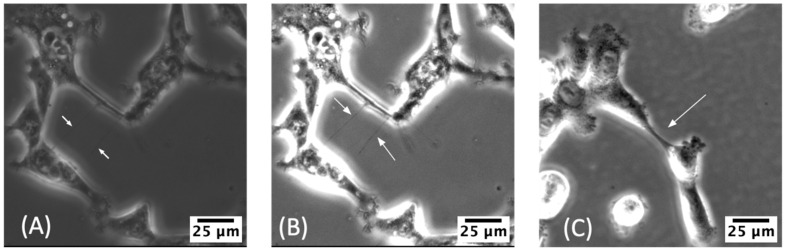
(**A**) Two TNTs that were successfully captured by the deep learning model (true positives). (**B**) The image from (**A**) is enhanced for improved TNT visibility. (**C**) A TNT-appearing structure that was mistakenly identified as a TNT by the model (false positive). Images (**B**,**C**) were generated with Fiji software and were adjusted for their brightness and contrast by setting minimum and maximum displayed value to 20 and 100, respectively, for improved visibility of the structures (this image modification is not necessary for the deep learning model to work).

We used the free version of Google Colab with hardware specifications of 12–14 GB of RAM, a CPU of Intel^®^ Xeon^®^ at 2.20 GHz, 30–35 GB of available disk space and Nvidia K80/T4 GPU with 12GB/16GB RAM (https://colab.research.google.com/drive/151805XTDg--dgHb3-AXJCpnWaqRhop_2, last accessed: 5 July 2022). Figure 1 depicts the possible outcomes of ML algorithms, the detection of TNTs (Figure 1A,B), and the mislabeling other cellular features as TNTs (Figure 1C). Due to the presence of noise, uneven illumination, irregular cellular shapes, and thinness of TNT lines with respect to cellular membranes, the visibility of TNTs is significantly reduced. TNTs are surrounded by darker intercellular regions, and occasionally the TNT lines become invisible, merging with the darker background. Our method is implemented on 2D phase-contrast images and consists of three main components: a preprocessing step to prepare the dataset in terms of de-noising the dataset and enhancing label quality; a sequence of two deep learning models to detect the TNTs; and a final step to count the TNTs and cells to provide a measure of the TNT-to-cell ratio (TCR) in the images. The TCR metric is essentially the same as our previous reports using the term ‘TNT Index’ to indicate the average number of TNTs per cell across multiple fields of view for a given set of cell culture conditions [45,53,54]. The TCR or TNT index can be used to monitor changes in cell culture over time and/or following drug exposure or other forms of treatment [45,53,54].

### 3.2. Pre-Processing

#### 3.2.1. Removal of Tile Shadows

**Figure 2:**
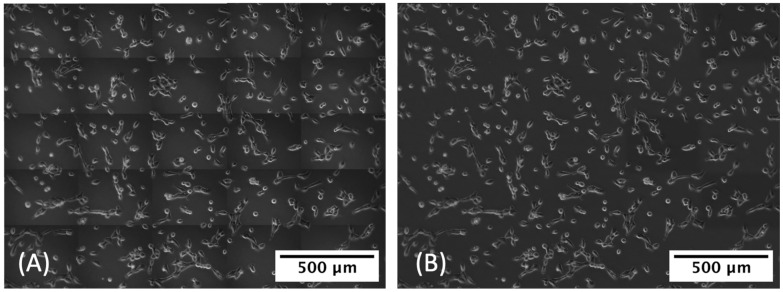
(**A**) Tiled image with shadows at edges of the tiles and (**B**) the same image with the shadows removed to prevent a high false-positive detection rate.

The original images were created by taking a grid of 5 × 5 tiled images, each measuring 1388 × 1040 pixels, and then stitching them together (Figure 2). This process resulted in shadows along the stitched edges, which significantly degraded the model performance at later stages. To remove those shadows, we used BaSiC, an image correction method for background and shading correction for image sequences, available as a Fiji/ImageJ plugin [73].

#### 3.2.2. Label Correction

To train an automated model, it is critical to obtain accurately labeled TNTs on the images in the training set. Since TNTs will be automatically identified pixel wise in later stages of the model, it is essential to label the TNTs in fine detail. However, when labeling visible TNTs, the human-marked TNTs are not fully capturing the width of the TNTs pixel wise. This, in turn, degrades model performance.

**Figure 3:**
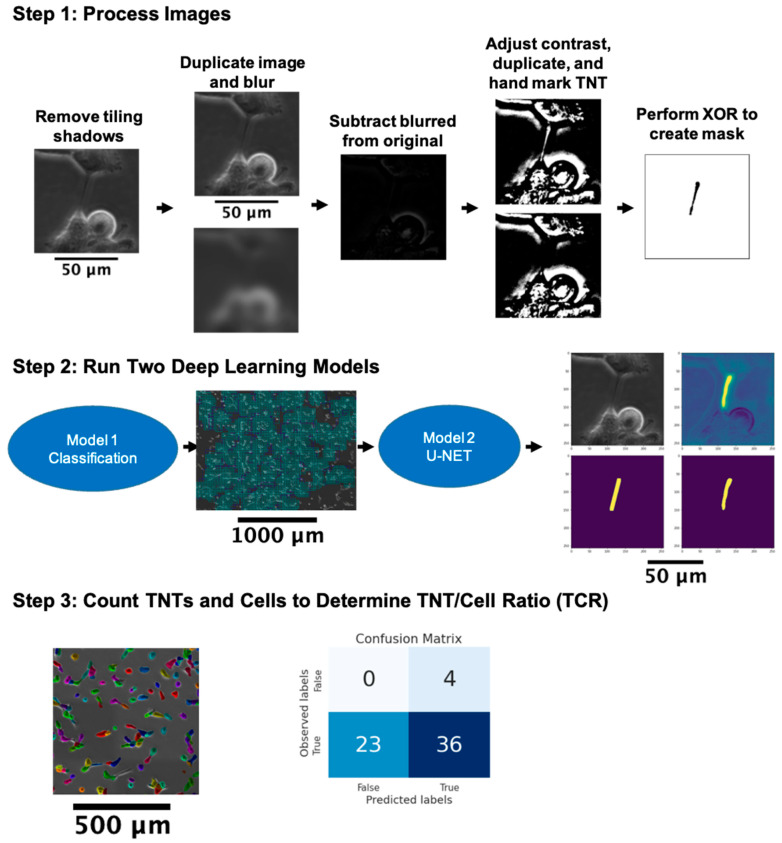
Flow diagram of AI-based TNT detection. Images were (Step 1) pre-processed for label correction and (Step 2) subdivided into a matrix of smaller image regions (‘patches’) that were classified as either containing or not containing any TNT structures, and pixel-wise classified regarding whether each pixel belonged to a TNT structure or not (see Supplementary Figures S1 and S2 and Supplementary Table S2). In (Step 3), the numbers of TNTs and cells were counted, and the TNT-to-cell ratio (TCR) was calculated (each colored object is an individual cell) and confusion matrix was reported (see Table 2 and Supplementary Table S3). XOR = bitwise exclusive or operator.

Figure 3 Step 1 shows the general outline of the preprocessing workflow, not including the removal of stitching shadows that is shown in Figure 2. To improve the quality of the labels on an image, two copies of that image are created. One of the copies is deblurred using Richardson-Lucy deconvolution with a Gaussian kernel of 7 × 7 and a standard deviation of 20 [74,75]. The deblurred copy is then subtracted from the original image. The resulting image is turned into a black and white 8-bit binary format and is once again duplicated. In one of these images, all visible TNTs, including their entire width, are colored with black ink. An XOR (bitwise exclusive or) operation is performed between the TNT-marked image and the duplicate unmarked image. The resulting image yielded the TNT masks [76].

### 3.3. Detecting TNT Regions

This section introduces our deep learning pipeline approach to detect and count TNTs.

#### 3.3.1. Classifying TNT-Inclusive Regions

**Figure 4:**
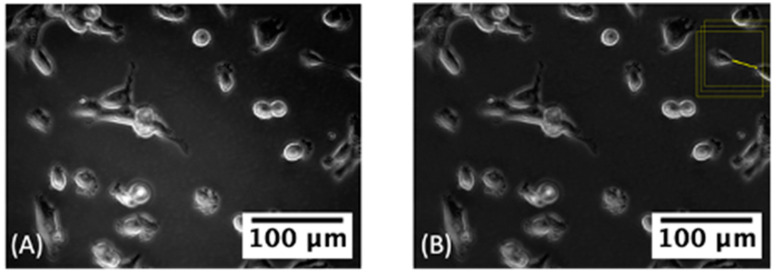
(**A**) Original image containing large pockets of TNT-free spaces. (**B**) After correcting edge artefacts as shown in Figure 2, the TNT-containing “patches” (yellow squares) showed where TNTs were captured within the matrix of smaller image regions. See Supplementary Figures S1 and S2.

With respect to the total area of an image, TNTs constitute a smaller percentage of the pixels. We approached the TNT detection problem in two steps: First, we trained a deep learning based classification model to rule out the large pockets of TNT-free spaces in the images. Our aim was to reduce the computational burden of detecting and segmenting TNTs in the next step, where we trained a second deep learning model to identify the TNT pixels (Figure 3 Step 2). The first step in our method also helped us break a single large image into smaller pieces and thus increased training data points for our models (Figure 4, Supplementary Figures S1 and S2).

The original images in the training dataset were stitched together resulting in an image size of 6283 × 4687 pixels. The images were then scanned with a sliding window of size 512 × 512 pixels with a stride of 10 pixels, extracting patches containing the TNT regions using a bounding box. The enclosed image region is then extracted. The area covered by the sliding window is labeled as “1” if there were a certain number of prelabeled TNT pixels within that window and also if those pixels were located closer to the center of the window. The reasoning behind checking whether the TNT pixels are closer to the patch center is to avoid partitioning of TNTs across sequential windows and thus losing the integrity of a TNT in a training data point. We repeat the same procedure with a sliding window size of 256 × 256 for images that are labeled as “1”. That is, we first identify the TNT including images with a bigger window, crop them from the original image, and then scan for TNTs with a smaller window inside the cropped images. Thus, we form two sets of images: a training set of 512 × 512 and another with 256 × 256. It is important to note that our method generated thousands of sub-images and sub-subimages from each of the four image sets studied here. For extensive details, please refer to the Supplementary Materials, Supplementary Figures S1 and S2, and Supplementary Table S2.

To train a classification algorithm to detect TNT-including images, we employed the VGGNet (16 layers) architecture, pre-trained on the ImageNet dataset [77]. Since the earlier layers of a pre-trained model are kept for learning the low-level image features, we replaced the VGGNet’s output layer with three hidden layers with 512, 170, and 70 nodes, respectively, and a binary output layer. We incrementally added these dense layers as we observed improvement in the performance of the classifier. To reduce overfitting, we also introduced a dropout layer of 60% dropout rate in between each pair of new fully connected layers. We trained two instances of this model, one for the images of size 512 × 512 pixels and another for those of size 256 × 256 pixels. We used the two models sequentially to identify image patches with TNTs. Only the images of size 256 × 256 pixels that included TNTs were fed into the U-Net model described below.

#### 3.3.2. U-Net with Attention Architecture for Segmentation

Since manual labeling of medical images is a labor-intensive and cumbersome task, automated medical image segmentation has been an active research area in the image-processing domain. After the advent of convolutional neural networks (CNN), many variants of CNN-based models have been proposed, which have advanced the state-of-the-art in image classification and semantic segmentation [78,79]. U-Net [6,80] is one of the commonly used architectures for medical image segmentation tasks due to its efficient use of graphics processing unit memory and superior performance [81]. In this study, we used a variant of U-Net, AURA-net [82], which uses U-Net with transfer learning to accelerate training and attention mechanisms to help the network focus on relevant image features.

U-Net is an encoder-decoder CNN-based architecture, which is composed of downsampling (encoder network) and upsampling (decoder network) paths. The encoder network, which is a contracting path, consists of the repeated application of two 3 × 3 convolutions, each followed by a rectified linear unit (ReLU) [83] and a 2 × 2 max pooling operation [84] with stride 2. At each step in the downsampling path, the number of feature channels is doubled. When running the encoder part, the model reduces the spatial dimensions of the image at every layer while capturing the features contained in the image with the help of filters.

The decoder network consists of layers, with each having (i) an upsampling of the feature map followed by a 2 × 2 up-convolution that halves the number of feature channels, (ii) a concatenation with the correspondingly cropped feature map from the encoder network side of the model, and (iii) two 3 × 3 convolutions, each followed by a ReLU. When training the decoder part of the model, the spatial aspect of the images are restored to make a prediction for each pixel in the image.

Although U-Nets are efficient in terms of training on a small number of data points, they can also benefit from transfer learning [82]. The usual transfer learning approach is to copy a certain number of layers from a pre-trained network to a target network to reduce the training time and increase model efficiency [85]. Next, we replaced the encoder network with the layers from a pre-trained ResNET model [86]. ResNET is trained on ImageNet [77], a set of natural images very different from the microscopic images in our study; however, the first layers of the ResNET model detects the features of images at a higher abstraction level, and thus, the transferred layers can be used to generalize these features for images from other contexts.

Attention-based models [87] are used to suppress less relevant regions in an input image and focus on more salient features relevant for the task. Attention U-Nets are shown to consistently improve the prediction performance of U-Net networks for various biomedical image segmentation tasks while preserving the model’s computational efficiency [81].

**Figure 5:**
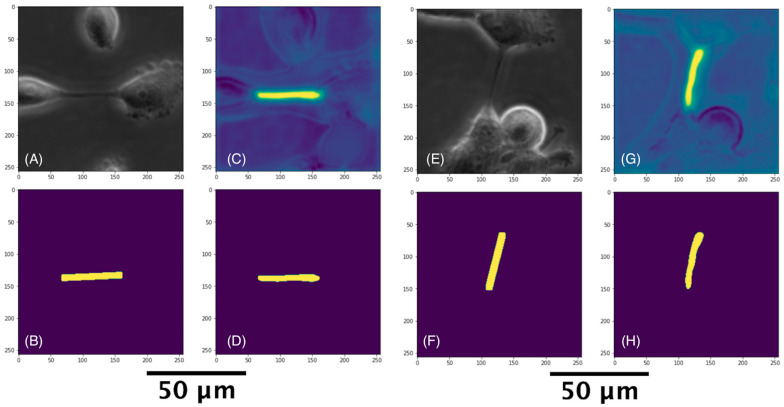
TNTs detected from two cropped images. (**A**,**E**) are the cropped raw images. (**B**,**F**) are the manually marked labels. (**C**,**G**) are the heatmap versions after prediction. (**D**,**H**) are the predicted TNTs.

We trained the U-Net model using the patches identified by the classification models described above. In training the models, we employed three loss functions, namely, binary cross-entropy (BCE) [88], Dice [89], and active contour (AC) loss [90]. Although Dice and BCE losses enforce the accuracy of predictions at the pixel level, the addition of AC loss allows consideration of area information. We adapted the use of these loss functions in our models from those used by Cohen and Uhlmann [82]. TNTs were segmented in the 256 × 256 pixel images by the U-Net model as shown in Figure 5. The AI-based model was able to recapitulate the human expert-based TNT identification.

### 3.4. Cell and TNT Counting

We used Cellpose, an anatomical segmentation algorithm [91], to count the number of cells in the images (Figure 3 Step 3). Cellpose utilizes a deep neural network with a U-Net style architecture and residual blocks, similar to the model used in this study for detecting TNTs. Moreover, Cellpose is trained on a dataset of various tissue types collected using fluorescence and phase contrast microscopy on different platforms, which made it an ideal candidate for this study.

To count TNTs, we first created an elliptical shaped kernel of size 5 × 5 pixels. We next performed a morphological transformation of the images, namely morphological gradient, which is the difference between the dilation and erosion of the structures in the images. Given the outline of the objects as an outcome of the transformation, we found the contours in the images, which are the curves joining contiguous points along a boundary between regions of different intensities. If the area of a contour was between 400 and 2500 pixels (44.89–280.56 µm^2^), it was counted as a TNT. We used OpenCV, an open-source library for computer vision, to process and analyze the images [92].

To evaluate the model performance, we used a separate test dataset that was not part of the training and tuning of the model. The “true” TNTs were those determined by consensus of the four human experts as described earlier.

The test image was partitioned into patches and then was fed sequentially into classification and U-Net models. Within each patch, a heatmap was generated. Next, the heatmaps of each patch were stitched together to form the overall heat map of the larger image. Following the counting rules described above, we counted and compared the number of TNTs predicted by the model vs. those identified manually by human experts (Table 1 and Supplementary Table S1). A pixel intensity threshold of 235 (range 0–255 in an 8-bit gray scale image) was chosen in the U-Net model because it maximized the sum of precision and recall (see Supplementary Figure S3). Our model was able to correctly identify 26.2% of the manually identified TNTs in the test dataset, whereas the identification rate was 49.9% for the test and training datasets combined. The precision for the test dataset was 41%. Our model generated more false-negative TNTs than false positive ones, hence a lower recall (sensitivity) compared to precision (positive predictive value). A few of the false positive TNTs were found to be true positives after double-checking the original images (see Supplementary Figure S4). Note that we report our performance evaluations without incorporating any adjustment for true positive numbers after double-checking. Next, we assessed the model’s ability to count predicted TNTs. For each image set, a human expert classified and counted the ML TNT predictions as FPs or TPs, and absence of ML TNT predictions as FNs, with respect to the human expert consensus “ground truth”. Supplementary Table S3 summarizes the human expert-based and ML-based counts. A fixed two-way ANOVA was performed (with factor 1 being the source of the count [i.e., human expert vs. ML model] and factor 2 being the image set evaluated [MSTO2-5]), using the F distribution (right-tailed). The results demonstrated no significant difference in human vs. ML counts of TNTs across the four image sets. For a detailed explanation of the three main reasons contributing to the generation of FPs and FNs by the model, see the end of the Supplementary Materials.

We next developed a new metric to measure the TNT-to-cell ratio (TCR) in the images (Table 2). We counted TNTs and cells and computed the number of TNTs per 100 cells (TCR × 100). A two-tailed t-test analysis determined there was no significant difference (*p* = 0.78) between the means of true and predicted TCRs.

## 4. Discussion

The detection and classification of cells have been active research areas for more than a decade [93]. There are various open-source and commercial software packages for cell counting and characterization for clinical and research purposes [94]; however, there is a dearth of specialized models for detecting TNTs. Here, we applied a precise quantitative analysis to construct an algorithm that uses the well-known U-Net deep learning model [6] to segment images and detect TNTs in vitro.

The main goal of this study was to present the fully automated end-to-end segmentation, detection, and counting process of TNTs. Even to a trained eye, it may be hard to decide whether a structure is a TNT or not. Therefore, it is a challenging task to develop an automated method to detect TNTs. As a result, automatic detection of TNTs has not been studied extensively. Hodneland et al. presented an automated method to detect nanotubes with a rule-based algorithm [76]. In their study, TNTs were identified by a series of transformations including watershed segmentation, edge detection, and mathematical morphology. Their method for cell segmentation was 3D, and they used two channels of cell images stained with two dyes. On the other hand, phase contrast microscopy is a label-free technique, making it well-suited for live-cell imaging without the need for a fluorescence microscope, which in turn makes the deep learning model presented here amenable to general use.

During the past decade, the field has evolved from reporting descriptions of TNTs and their cell morphology and function, to identifying changes in the numbers of TNTs over time. TNTs are dynamic structures that exist for minutes to hours [37,44,46,62]. We and others have previously demonstrated that they represent a form of cellular stress response to outside stimuli, including drug treatment and viral infection [38,49,52,53,95]. The identification of TNTs currently still rests on identification of morphologic characteristics that distinguish them from other cell protrusions, including invadopodia, filopodia, and lamellopodia [68,96,97,98]. However, quantitation is limited as the process is laborious without validated TNT-specific markers and relies currently on manual identification. AI-based approaches that could reliably identify TNTs with high specificity and sensitivity would move the field of TNT biology forward significantly by providing a new tool for rapid identification of TNTs and their fluctuation over time. We report the results using MSTO-211H cells in this manuscript at this early stage of our investigation into AI-based approaches for TNT detection, because this cell line has served as one of our optimal models for in vitro investigation of TNTs for over a decade. As we continue to build on this foundation of work, our next set of studies will utilize other cell lines, cancer and non-cancer, to further confirm and validate the model across diverse cell types.

Software programs have been developed previously to classify and quantify cellular features and colonies for the purpose of reliable automated forms of detection. Specific examples of this approach include evaluation of embryonic stem cell differentiation and pluripotency analysis [99]. Perestrelo et al. utilized mouse embryonic stem cells as a model for their software, Pluri-IQ [99]. Their software was able to quantify the percentage of pluripotent, mixed, or differentiated cells; it was also able to analyze different magnification image sizes and measure pluripotency by the markers that were used for evaluation. This group also showed the pipeline used for segmentation, machine training, validation, and finally automatic data comparison. Pluri-IQ can learn, based on colony morphology, how to evaluate according to the classifier pool where the colony’s best features fit when a new colony is put through the software [99].

Another software program, FiloDetect, is the first automated tool for detecting filopodia in cancer cell images [100]. Filopodia are long F-actin-based cellular protrusions whose primary purpose is to mediate cell motility. The FiloDetect approach has been evaluated in Rat2 fibroblasts and B16F1 mouse melanoma cell images and has been applied to measure the effects of PI4KIIIβ’s expression on filopodia production in BT549 breast cancer cells [100]. FiloDetect uses intensity-based thresholding with a combination of morphological operations [100]. It also uses additional processing to detect combined filopodia-filopodia that are fixed at the base or cross over at the length [100]. A similar filopodia-focused software program to highlight is FiloQuant [101]. This software is an Image J-based tool used to extract quantifiable data on filopodia dynamics, density, and length from both fixed and live-cell microscopy images [101]. It is able to be used in different cell types, microenvironments, and image acquisition techniques. It uses edge detection, intensity detection, and skeletonization via the AnalyzeSkeleton algorithm [101]. FiloQuant has a step-by-step user validation method to achieve optimal settings when identifying filopodia. By using this tool, filopodia formation and invasive capacity have been linked in 3D tumor spheroids [101]. This method was developed after researching the unique attributes and shortcomings of other filopodia identification techniques, such as FiloDetect, CellGeo [102], and ADAPT [103]. Each of these techniques lacks requirements for proprietary software, lacks customizable options for improvement, is able to analyze only single cells, does not have a density quantification tool, and is not easy to navigate for non-experts. FiloQuant overcomes these limitations [101].

The method we describe here in its current form has potential limitations. Some of the predicted TNT structures looked broken into pieces and this resulted in counting the same TNT multiple times. Our model consisted of two sequential classification models and needed careful calibration to identify TNTs. Reducing and simplifying our model to a single step is left for future studies. Importantly, TNTs are 3-dimensional protrusions that extend from one cell to another, or to other groups of cells. A well-established morphologic characteristic is their ability to ‘hover’ in the 3-dimensional plane when cultured in vitro under 2-dimensional cell culture conditions. Thus the most ideal conditions to characterize TNTs consist of high resolution imaging that permit 3D renderings by stacking images taken in the z-plane. However, for more routine assessment in 2D cell culture conditions, and considering the lack of a testable validated structural or compositional marker specific to TNTs, identification remains reliant on visual identification. TNTs comprise a heterogeneous group of cellular channels, displaying a relatively wide range of widths and lengths that may vary based on cell type of origin, underlying molecular machinery, and other yet unknown factors that remain to be elucidated. Challenges of automated identification include differentiation of some TNTs from more adherent forms of long protrusions, identification of established TNTs vs. those that are forming but not yet attached to recipient cells, separation from dense clusters amidst confluent or semi-confluent groups of cells, and other factors. Among other questions to be determined in future studies is whether AI-based forms of evaluation would work more optimally in cells imaged live, as compared to cells imaged following chemical or other fixation, which may introduce artefactual or other changes that have potential to disrupt the natural state of TNTs in vitro. The model presented here will evolve over time and is adaptable to address these and future needs.

Our AI-based TNT detection method, TNTdetect.AI, provides three principal contributions to the field. First, we propose a novel way to improve the manual labeling of TNTs, which would help pixel-wise detection of TNTs. Second, we can sequentially train two classification models to detect TNTs, including regions and image pixels representing the TNTs, and third, we propose a new metric to quantify TNT intensity in an image, namely, the TNT-to-cell ratio (TCR). This metric can be used in evaluating, for example, the impact of treatments on cancer cells by capturing TCRs at different stages of therapy. Our automated TNT detection approach is different from Hodneland et al.’s method in two ways. First, we created a deep learning-based model that does not require the definition of if-then rule statements. Second, we trained our model with a single information channel, 2D phase contrast microscopy images.

## 5. Conclusions

In summary, we report the application of TNTdetect.AI, an automated model generated by deep learning and trained to label and detect TNTs and cells imaged in culture. The continued application and refinement of this process will provide a new approach to the analysis of TNTs, which form to connect cancer and other cells. This approach has the potential to enhance the drug screens intended to assess therapeutic efficacy of experimental agents, and to reproducibly assess TNTs as a potential biomarker of response to therapy in cancer.

## Figures and Tables

**Table 1:** Results of TNT detection for three training sets and one test set. FP = false positive, PPV = positive predictive value. * True, identified by human experts. f-1 score = 2 × [PPV × sensitivity]/[PPV + sensitivity].

Image Set	No. of TNTs (True *)	PPV (Precision)	Sensitivity (Recall)	No. of FPs	No. of Human Expert-Corrected FPs	f-1 Score
Training 1 (stitched image MSTO2)	43	0.67	0.70	14	0	0.68
Training 2 (stitched image MSTO3)	18	0.38	0.61	17	1	0.47
Training 3 (stitched image MSTO4)	33	0.52	0.42	13	1	0.47
Test 1 (stitched image MSTO5)	42	0.41	0.26	16	2	0.32

**Table 2:** Results reporting the tunneling nanotube (TNT)-to-cell ratio (TCR, or TNT index). * True, identified by human experts. ** Predicted, detected by the model.

Image Set	No. of TNTs (True *)	No. of TNTs (Predicted **)	No. of Cells (from Cellpose)	TCR × 100 (True *)	TCR × 100 (Predicted **)
Training 1 (stitched image MSTO2)	43	45	897	4.79	5.02
Training 2 (stitched image MSTO3)	18	29	777	2.32	3.73
Training 3 (stitched image MSTO4)	33	27	754	4.38	3.58
Test 1 (stitched image MSTO5)	42	27	897	4.68	3.01

## Data Availability

The data that support the findings of this study are available from the corresponding authors, E.L. and C.B.P., upon reasonable request.

## Supplementary Materials

The following supporting information can be downloaded at: https://www.mdpi.com/article/10.3390/cancers14194958/s1, Figure S1: Each stitched image of MSTO-211H cells was subdivided into subimages (dark blue colored squares of size 512 × 512 pixels) or sub-subimages (cyan colored squares of size 256 × 256 pixels) via a sliding window. This allowed for one stitched image to generate thousands of subimages upon which the machine learning model could be trained.; Figure S2: the size of the patches (number of rows × number of columns within each patch) within the MSTO-211H cell stitched and padded images of size 6795 × 5199 pixels; Figure S3: the values of precision and recall as a function of varying the pixel intensity threshold (range 0–255) in Model 2 (U-Net). It shows that the pixel intensity threshold of 235 maximized the sum of precision and recall; Figure S4: illustrates circumstances in which there was agreement or disagreement between the identification of structures as TNTs, between the human expert consensus and the ML model.; Table S1: Results of inter-rater agreement for TNT identification in stitched MSTO-211H images among the four human experts, using the Cohen’s kappa statistic.; Table S2: Number of training subimages generated from each stitched image (image set) of MSTO-211H cells, used for Model 1; Table S3: Tabulated summary of the human expert-based and ML-based TNT counts. For each image set, a human expert classified and counted the ML TNT predictions as FPs or TPs, and absence of ML TNT predictions as FNs, with respect to the human expert consensus “ground truth”. Ref. [104] is cited in the Supplementary Materials.

## Abbreviations

AC	active contour
AI	artificial intelligence
AURA-net	modified U-Net architecture designed to answer the problem of segmentation for small datasets of phase contrast microscopy images
BCE	binary cross-entropy
CNN	convolutional neural network
FP	false positive
ML	machine learning
OpenCV	open-source computer vision library
PPV	positive predictive value
ReLU	rectified linear unit
ResNET	residual neural network
TCR	TNT-to-cell ratio
TNT	tunnelling nanotube
U-Net	a convolutional network architecture for fast and precise image segmentation
VGGNet	a convolutional neural network architecture to increase ML model performance
