# Supplementary material for: TNTdetect.AI: A Deep Learning Model for Automated Detection and Counting of Tunneling Nanotubes in Microscopy Images
Source: Cancers (Basel). 2022 Oct 10;14(19):4958. doi: 10.3390/cancers14194958 (PMC9562025; doi:10.3390/cancers14194958)
Supplement: Supplementary file 1 [file cancers-14-04958-s001.zip › cancers-1781504-Supplementary Material.pdf]

SUPPLEMENTARY MATERIAL (Methods, Results, and References)

TNTdetect.AI: A deep learning model for automated detection and counting of tunneling nanotubes in microscopy images

Yasin Ceran, Hamza Ergüder, Katherine Ladner, Sophie Korenfeld, Karina Deniz, Sanyukta Padmanabhan, Phillip Wong, Murat Baday, Thomas Pengo, Emil Lou and Chirag B. Patel

Supplementary Methods and Results

Currently because there is no known biomarker for TNTs, the process of identifying TNTs on images is, by definition, subjective. Some TNTs are obvious, and easily found even by a non-expert. However there are many other TNTs that are not obvious and require experience to identify. This is due to multiple factors including the resolution or magnification of an image, the fact that TNTs are dynamic (lengthening, breaking, starting as multiples and trimming or converging to one) and microscopy images catch them at a moment in time. Their appearance also varies widely between cell types in length, thickness, uniformity and quantity. Given all the variability of TNTs, it takes time for the human eye to be trained to classify a cellular extension as a TNT. For this study, four members of our lab, "TNT identification human experts", independently identified and labeled TNTs in a given image. However after the first image, we realized that they needed to review the rules they each used to make a positive identification within the cell line under study (MSTO-211H).

These rules included: only count TNTs that connect two cells, are not too thick, and are easily visible. Reviewing rules helped with the identification, but in the end the decisions were still subjective. Individually, on their own computer set-ups, the human experts had to decide the following questions: is this extension too thick?, are these cells really touching?, and is this a shadow, a stress fiber, or a real TNT? The most discrepancies came with thin, faint, or double extensions. After the four experts independently labeled TNTs on four stitched images each, for each image, the structures identified as TNTs were compared across the four experts. A tally was created to determine how many reviewers agreed that a structure was a TNT. If three or four experts identified a structure as a TNT, then it was considered a TNT by human consensus (majority rule). If two experts identified a structure as a TNT ("tie"), then all four experts collectively reviewed the structure to adjudicate whether the human consensus was for or against labeling the structure a TNT. Finally, if only one expert identified a structure as a TNT, then it was by default considered to not be a TNT by human consensus. However, there was occasionally an exception to this rule, which occurred when a single expert identified a structure they thought was a TNT located at the edge of the stitched image or within a clump of cells. All four experts collectively reviewed the structure to adjudicate whether it would be reconsidered from the default assessment (not a TNT) to an updated human consensus of the structure being a TNT. The results are summarized in **Supplementary Table S1**. This process reflects the challenges of purely objective TNT identification by human experts, and therefore the need for a deep learning-based algorithm to perform and quantitate TNT detection.

Table S1. Results of inter-rater agreement for TNT identification in stitched MSTO-211H images among the four human experts, using the Cohen's kappa statistic.

Image set	No. of TNTs identified by each human expert, total no. of unique TNTs identified across all experts, and consensus no. of TNTs	Cohen's kappa (human expert inter-rater agreement)	Level of agreement	Z	p-value
Training 1 (stitched image MSTO2)	Expert 1 no. TNTs: 41<br>Expert 2 no. TNTs: 41<br>Expert 3 no. TNTs: 43<br>Expert 4 no. TNTs: 40<br>Total unique TNTs: 52<br>Consensus number: 43	0.23	Fair	4.03	$p<0.00001$
Training 2 (stitched image MSTO3)	Expert 1 no. TNTs: 20<br>Expert 2 no. TNTs: 19<br>Expert 3 no. TNTs: 22<br>Expert 4 no. TNTs: 20	0.45	Moderate	5.36	$p<0.00001$

	Total unique TNTs: 24<br>Consensus number: 18				
Training 3 (stitched image MSTO4)	Expert 1 no. TNTs: 32<br>Expert 2 no. TNTs: 22<br>Expert 3 no. TNTs: 29<br>Expert 4 no. TNTs: 31<br>Total unique TNTs: 37<br>Consensus number: 33	0.19	Slight	2.76	p=0.0029
Test 1 (stitched image MSTO5)	Expert 1 no. TNTs: 47<br>Expert 2 no. TNTs: 38<br>Expert 3 no. TNTs: 46<br>Expert 4 no. TNTs: 45<br>Total unique TNTs: 55<br>Consensus number: 42	0.33	Fair	5.85	p<0.00001

We used four image sets in this study (n=3 training image sets [MSTO2, MSTO3, and MSTO4] and n=1 test image set [MSTO5]). Each image set contained a stitched image of 5 × 5 smaller microscopy images (tiles) of the MSTO cells that was further subdivided into thousands of smaller images (called subimages or sub-subimages). Each of the 25 tiles making up the stitched image was 1,388 × 1,040 pixels in size. The neighboring tiles were stitched together after calculating the similarity between the overlapping regions at the edges [104]. Since overlapping regions from two tiles were combined into a single stitched region, the length and width of the resulting stitched image were less than 5 times the length and width of a single tile. For the purposes of this example, we refer to image set MSTO2. The resulting image was referred to as a stitched image (with size 6,283 × 4,687 pixels). The stitched image was padded with 256 pixels on all four sides, by reflection. The four corners (size 256 × 256 pixels each) were created by reflecting the adjacent 256 × 256 pixels from the newly created horizontal reflections above and below the original stitched image. The purpose of the padding was to ensure that, during the 512 × 512 pixel-sized sliding window process described below, any TNTs at the edges of a subimage could be detected. This resulted in the final stitched and padded image size of 6,795 × 5,199 pixels.

Furthermore, each stitched image was subdivided into subimages (dark blue colored squares of size 512 × 512 pixels) or sub-subimages (cyan colored squares of size 256 × 256 pixels) via a sliding window, as described below and shown in **Supplementary Figure S1** below.

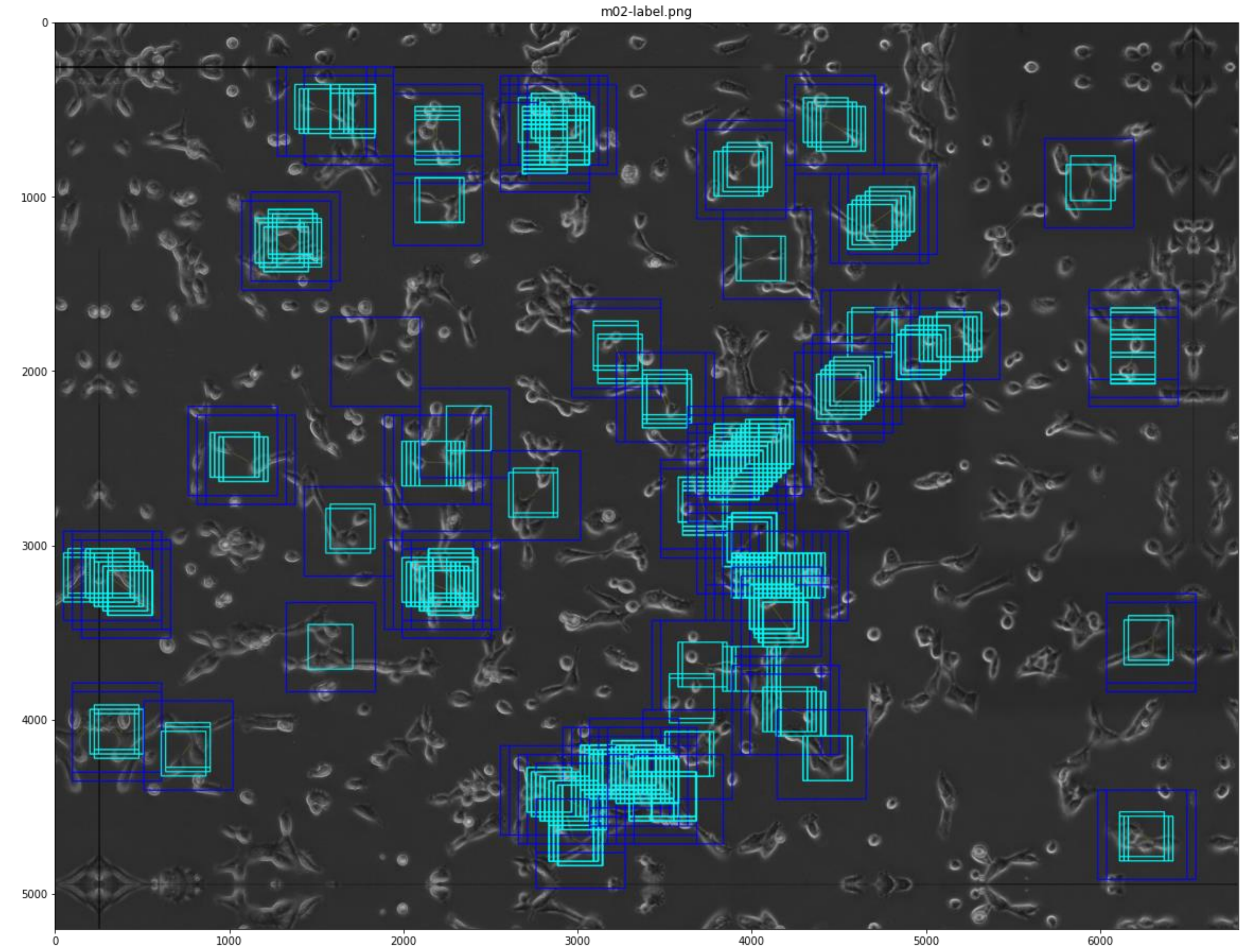

Figure S1. Each stitched image of MSTO-211H cells was subdivided into subimages (dark blue colored squares of size 512 × 512 pixels) or sub-subimages (cyan colored squares of size 256 × 256 pixels) via a sliding window. This allowed for one stitched image to generate thousands of subimages upon which the machine learning model could be trained.

This improved the performance of the ML model because it allowed for hundreds of training data points to be entered into Model 1 (TNT classification) and Model 2 (U-NET, TNT detection). Model 1 was trained on subimages of size 512 × 512 pixels to detect regions of each stitched image that contained a TNT (defined as connecting two cells). Starting with the upper left-hand corner of each stitched image, it was divided into 154 non-overlapping patches (n=130 patches of size 512 × 512 pixels each, n=10 patches of size 139 × 512 pixels each, n=13 patches of size 79 × 512 pixels each, and n=1 patch of size 79 × 139 pixels). **Supplementary Figure S2** below shows the size of the patches (number of rows × number of columns within each patch) within the stitched and padded image of size 6,795 × 5,199 pixels.

512 × 512	512 × 512	512 × 512	512 × 512	512 × 512	512 × 512	512 × 512	512 × 512	512 × 512	512 × 512	512 × 512	512 × 512	512 × 512	512 × 139
512 × 512	512 × 512	512 × 512	512 × 512	512 × 512	512 × 512	512 × 512	512 × 512	512 × 512	512 × 512	512 × 512	512 × 512	512 × 512	512 × 139
512 × 512	512 × 512	512 × 512	512 × 512	512 × 512	512 × 512	512 × 512	512 × 512	512 × 512	512 × 512	512 × 512	512 × 512	512 × 512	512 × 139
512 × 512	512 × 512	512 × 512	512 × 512	512 × 512	512 × 512	512 × 512	512 × 512	512 × 512	512 × 512	512 × 512	512 × 512	512 × 512	512 × 139
512 × 512	512 × 512	512 × 512	512 × 512	512 × 512	512 × 512	512 × 512	512 × 512	512 × 512	512 × 512	512 × 512	512 × 512	512 × 512	512 × 139
512 × 512	512 × 512	512 × 512	512 × 512	512 × 512	512 × 512	512 × 512	512 × 512	512 × 512	512 × 512	512 × 512	512 × 512	512 × 512	512 × 139
512 × 512	512 × 512	512 × 512	512 × 512	512 × 512	512 × 512	512 × 512	512 × 512	512 × 512	512 × 512	512 × 512	512 × 512	512 × 512	512 × 139
512 × 512	512 × 512	512 × 512	512 × 512	512 × 512	512 × 512	512 × 512	512 × 512	512 × 512	512 × 512	512 × 512	512 × 512	512 × 512	512 × 139
512 × 512	512 × 512	512 × 512	512 × 512	512 × 512	512 × 512	512 × 512	512 × 512	512 × 512	512 × 512	512 × 512	512 × 512	512 × 512	512 × 139
512 × 512	512 × 512	512 × 512	512 × 512	512 × 512	512 × 512	512 × 512	512 × 512	512 × 512	512 × 512	512 × 512	512 × 512	512 × 512	512 × 139
79 × 512	79 × 512	79 × 512	79 × 512	79 × 512	79 × 512	79 × 512	79 × 512	79 × 512	79 × 512	79 × 512	79 × 512	79 × 512	79 x 139

Figure S2. shows the size of the patches (number of rows × number of columns within each patch) within the MSTO-211H cell stitched and padded images of size 6,795 × 5,199 pixels.

The patches were displaced as sliding windows of size 512 × 512 pixels, in increments of 51 pixels per displacement along the horizontal or vertical direction; these overlapping regions of size 512 × 512 pixels that were generated from each patch were referred to as subimages. The consecutive subimages in any one direction overlapped by 461 × 512 pixels. Starting with the upper left-most pixel in each patch (row 1, column 1), it was displaced by the sliding window in n=11 horizontal steps (no step [home position] + 51 pixels/step) and n=11 vertical steps (no step [home position] + 51 pixels/step), thereby generating n=121 subimages of size 512 × 512 pixels from each of the 108 patches of size 512 × 512 pixels (colored in white in **Supplementary Figure S2**), or a total of 13,068 subimages. Because the remaining 22 patches of size 512 × 512 pixels at the right and bottom sides (colored in gray in **Supplementary Figure S2**) were bordered outside by patches smaller than 512 × 512 pixels, they generated a total of 567 patches). The patches on the right-most (colored in yellow in **Supplementary Figure S2**) and bottom-most (colored in green in **Supplementary Figure S2**) sides of each original stitched image were each less than 512 × 512 pixels in size (i.e., 512 × 139 pixels [n=10], 79 × 512 pixels [n=13], or 79 × 139 pixels [n=1]), they generated 237 subimages. Therefore, this window sliding procedure resulted in a total of 13,872 subimages.

Within each patch, the subimages that contained a structure at the middle (i.e., not at an edge) that was predicted to be a TNT were labeled as "1", else they were labeled as "0". We needed subimages labeled as 0 (along with those labeled as 1) to train Model 1 (TNT classification). Therefore, we sampled n=4 subimages per patch, specifically the second and seventh subimages in the top row (moving from left-to-right) and the second and seventh subimages in the left-most row (moving from top-to-bottom) within each patch; the selection of second and seventh was arbitrary. From the 154 patches of various sizes, n=516 subimages were sampled (n=432 from the n=108 patches of size 512 × 512 pixels, and the remaining n=84 sampled subimages from the remaining n=46 patches of smaller size). Some of the sampled subimages might have contained a structure predicted to be a TNT and therefore would have already been labeled as 1. Thus, 406 subimages were sampled in image set MSTO2. **Supplementary Table S2** below shows the number of training subimages used for Model 1.

Table S2. Number of training subimages generated from each stitched image (image set) of MSTO-211H cells, used for Model 1. *Labeling of the subimages for the first part of Model 1 was "1" if they contained a structure at the middle (i.e., not at an edge) that was predicted to be a TNT, and "0" otherwise. **Labeling of the sub-subimages for Model 2 was "1" if any of the pixels in them were predicted to be part of a TNT and "0" otherwise.

Image set	No. overlapping training subimages for firs part of Model 1 (TNT classification) generated by sliding windows of size 512 × 512 pixels*	No. sub-subimages (size 256 × 256 pixels) generated from subimages labeled as "1" (400 sub-subimages per subimage)	No. training sub-subimages for Model 2 (U-NET, TNT detection) generated by sliding windows of size 256 × 256 pixels**
Training 1 (stitched image MSTO2)	406<br>(n=290 labeled "0", n=116 labeled "1")	46,400	1,725<br>(n=740 labeled "0", n=985 labeled "1")
Training 2 (stitched image MSTO3)	449<br>(n=387 labeled "0", n=62 labeled "1")	24,800	955<br>(n=423 labeled "0", n=532 labeled "1")
Training 3 (stitched image MSTO4)	414<br>(n=342 labeled "0", n=72 labeled "1")	28,800	1,020<br>(n=546 labeled "0", n=474 labeled "1")
Test 1 (stitched image MSTO5)	424<br>(n=319 labeled "0", n=105 labeled "1")	42,000	1,641<br>(n=668 labeled "0", n=973 labeled "1")

Model 2 (U-NET, TNT detection) was trained on sub-subimages of size 256 × 256 pixels, to detect whether each pixel was part of a TNT or not (pixel-by-pixel analysis). The sub-subimages of size 256 × 256 pixels were generated as follows. Within each of the subimages of size 512 × 512 pixels (labeled as 1 or 0) selected to train Model 1 (TNT classification), we next repeated a window sliding procedure similar to the initial procedure used to generate the subimages from each tile. The sub-subimages generated from the subimages were 256 × 256 pixels in size. In **Supplementary Figure S1** above, the cyan colored boxes indicate sub-subimages of 256 × 256 pixels in size that were generated by the second sliding window, which moved in 25-pixel step sizes (thereby generating 400 sub-subimages from each subimage labeled as "1"). It should be noted that there were multiple overlapping cyan colored subimages corresponding to a single TNT. Only subimages (of size 512 × 512 pixels) labeled as "1" in the first part of Model 1 were used to generate sub-subimages. For purposes of training the second part of Model 1 and TNT detection in Model 2, each sub-subimage was labeled as "1" if it contained any portion of a TNT (unlike the requirement of labeling "1" for the subimages, which was that they contained a predicted TNT at the middle), and labeled as "0" otherwise. In image set MSTO2, from n=406 overlapping subimages that were used as the training dataset for the first part of Model 1, there were 46,400 sub-subimages of size 256 × 256 pixels that were generated to train the second part of Model 1. Among the 46,400 sub-subimages, n=985 were labeled as "1" and the remaining ones were labeled as "0". Using a similar procedure to the subimage sampling outlined above, we next performed a sub-subimage sampling procedure to include some sub-subimages labeled "0" in the training data set for Model 2. The last column of **Supplementary Table S2** above summarizes the number of sub-subimages (labeled as "1" or "0") that were ultimately used to train Model 2, which identified TNTs on a pixel-by-pixel basis.

Because this study aimed to detect structures that were TNTs (i.e., its purpose was not to detect the absence of TNTs), the ML model could only identify true positives (TPs), not true negatives (TNs). Due to the emphasis of the ML model on positively detecting TNTs, we created precision and recall curves as a function of the threshold of pixel intensity in Model 2 that was used to determine whether a pixel was contained in a TNT or not; a receiver-operator curve (ROC) could not be created. The threshold changes between 0 and 255, the range of pixel intensities in an 8-bit gray scale image. **Supplementary Figure S3** below shows the values of precision and recall as a function of varying the pixel intensity threshold (range 0-255) in Model 2. It shows that the pixel intensity threshold of 235 maximized the sum of precision and recall.

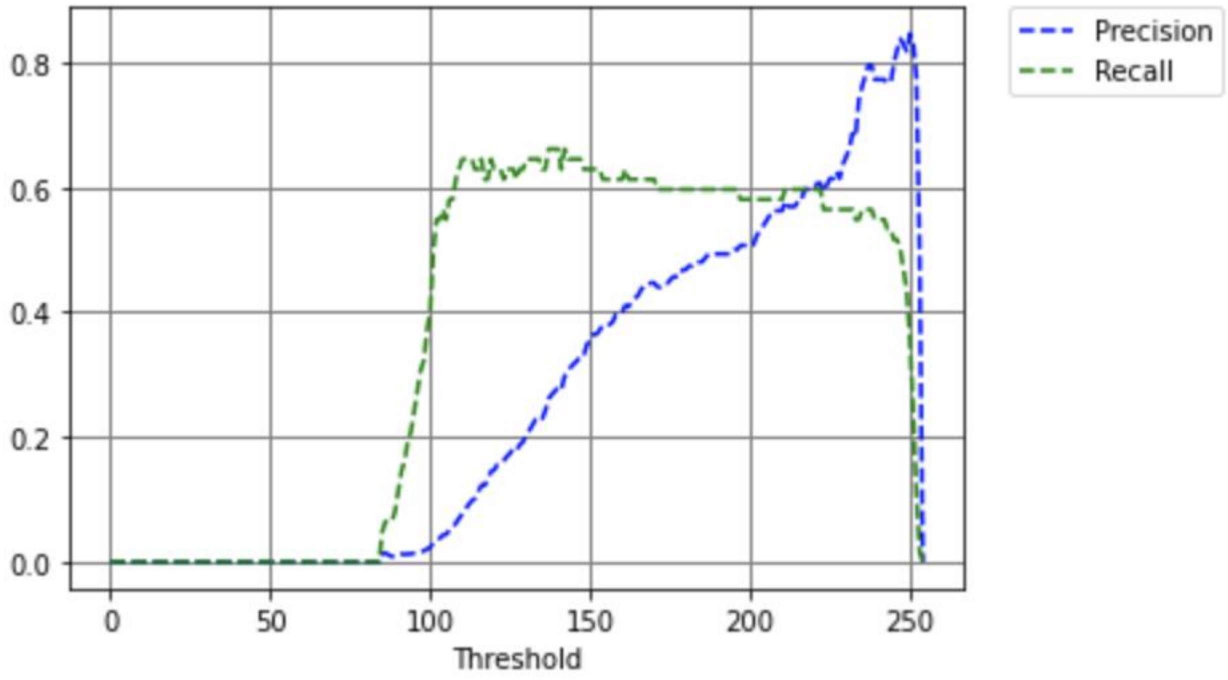

Figure S3. shows the values of precision and recall as a function of varying the pixel intensity threshold (range 0-255) in Model 2 (U-Net). It shows that the pixel intensity threshold of 235 maximized the sum of precision and recall.

We next performed a head-to-head comparison of the human expert consensus identification of TNTs against that of the raw output of the ML model (based on a pixel intensity threshold of 235 and total pixel area threshold of 400 pixels). This allowed us to create contingency tables containing the tallies of true positives (TPs), false positives (FPs), and false negatives (FNs). Based on these numbers, we were able to calculate sensitivity (also referred to as recall: [TP / (TP + FN)]), positive predictive value (PPV, also referred to as precision: [TP / (TP + FP)]), and the F1 score (2 × [PPV × sensitivity] / [PPV + sensitivity]). The results are shown in **Table 1** in the main manuscript.

Supplementary Figure S4 below illustrates circumstances in which there was agreement or disagreement between the identification of structures as TNTs, between the human expert consensus and the ML model. The TNTs identified by human expert consensus are colored in yellow and the TNTs identified by the ML model are colored in blue. Any structures that were identified as a TNT by at least one human expert during independent review, but that were ultimately decided by human expert consensus to not be a TNT, are colored in red. **Supplementary Figures S4a-S4d** are from one of the training image sets (stitched image MSTO2). **Supplementary Figure S4a** is an example of a false negative (FN, @ symbol) in which the ML model missed a TNT that was identified by consensus of the human experts. **Supplementary Figure S4b** is an example of four true positives (TPs) of short and long TNTs identified by human expert consensus that were also identified as TNTs by the ML model; one of the TNTs was only partially identified by the ML model (* symbol). This partial identification can still have utility at this stage of development of the ML model, in which follow-up human expert review is still required to consider whether or not the initial consensus decision should be changed or not. **Supplementary Figure S4c** is an example of a TP (* symbol) in which the ML model correctly identified a TNT determined by human expert consensus; a pseudo "true negative" (TN, # symbol) in which the structure was identified by a human expert to be a TNT during independent review but was ultimately decided to not be a TNT by subsequent human expert review and consensus, and which was also not identified as a TNT by the ML model – it is important to note that there are no pure TNs in this study because the human experts and ML model were only tasked with identifying the presence (not the absence) of TNTs; a FN (@ symbol) in which the human expert consensus identified a TNT but it was missed by the ML model; and a FP (^ symbol) in which the ML model mis-labeled the edge of a cell as a TNT. **Supplementary Figure S4d** demonstrates the similarities in human vs. ML identification of TNTs as well as the utility of the ML approach to aid human experts. In the upper left-hand corner, a FP (^ symbol) is seen in which at least one human expert independently identified the structure as a TNT but it was ultimately decided not to be a TNT by human expert consensus. In this case, the ML model identified a portion, but not the full length, of the structure to be a TNT. Although this is considered a FP, it highlights the challenges that both human experts and ML models face in regard to identifying TNTs. In the lower right-hand corner, the structure was originally identified as a FP (^^ symbol) because it was not identified as a TNT by human expert consensus. However, after the human experts reviewed the results of ML-based TNT labeling, they re-classified the structure as indeed being a TNT. This is an example of the ML

model being able to assist the human experts in identifying structures that may be TNTs, particularly at the edges of images. **Supplementary Figure S4e** is from the test image set (stitched image MSTO5). Similar to **Supplementary Figures S4c and S4d**, it shows an example of TP (* symbol) TNT identified partially along its length by the ML model and two FPs (@ symbol) that are long intersecting TNTs identified by human expert consensus but missed by the ML model. **Supplementary Figure S4e** also shows two structures that were originally identified as FPs (^^ symbol) because they were not identified as TNTs by human expert consensus. However, after the human experts reviewed the results of ML-based TNT labeling, they re-classified the structures as indeed being TNTs. These are other examples of the ML model being able to assist the human experts in identifying structures that may be TNTs, particularly at the edges of images.

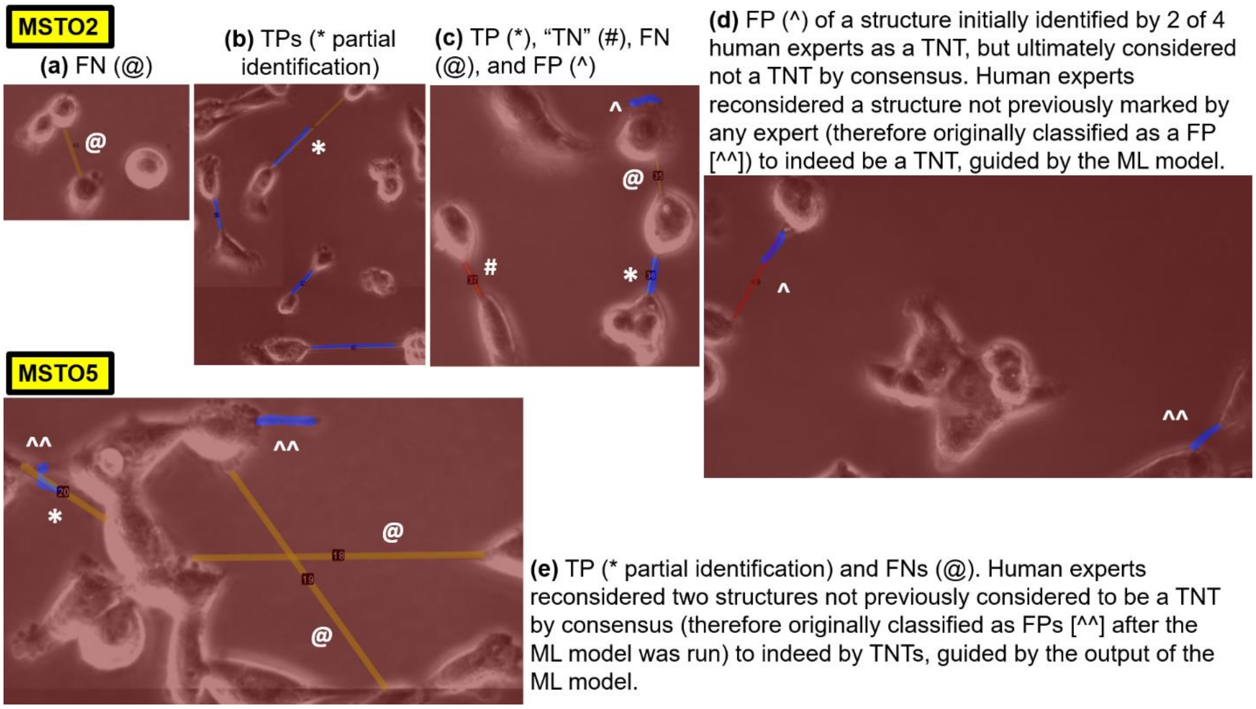

Figure S4. illustrates circumstances in which there was agreement or disagreement between the identification of structures as TNTs, between the human expert consensus and the ML model. See above for detailed explanation.

Next, we assessed the model's ability to count predicted TNTs. For each image set, a human expert classified and counted the ML TNT predictions as FPs or TPs, and absence of ML TNT predictions as FNs, with respect to the human expert consensus "ground truth". **Supplementary Table S3** below summarizes the human expert-based and ML-based counts. A fixed two-way ANOVA was performed (with factor 1 being the source of the count [i.e. human expert vs. ML model] and factor 2 being the image set evaluated [MSTO2-5]), using the F distribution (right-tailed). The results demonstrated no significant difference in human vs. ML counts across the four image sets (factor 1 F statistic 0.14, 1 degree of freedom, p=0.71; factor 2 F statistic 0.37, 3 degrees of freedom, p=0.78; interaction term of factor 1 * factor 2 F statistic 0.02, 3 degrees of freedom, p=0.995).

Table S3. Tabulated summary of the human expert-based and ML-based TNT counts. For each image set, a human expert classified and counted the ML TNT predictions as FPs or TPs, and absence of ML TNT predictions as FNs, with respect to the human expert consensus "ground truth".

Parameter	Image set	Human expert count	ML count (see Step 3 of Figure 3 in the main manuscript)
False positives	Training (MSTO2)	15	10
	Training MSTO3	18	17
	Training MSTO4	13	8
	Test (MSTO5)	16	12
True positives	Training (MSTO2)	30	35
	Training MSTO3	11	12
	Training MSTO4	14	18

	Test (MSTO5)	11	13
False negatives	Training (MSTO2)	13	27
	Training MSTO3	7	16
	Training MSTO4	19	22
	Test (MSTO5)	31	36

There are three main reasons why the model generated false positive and negatives:

1. There are hyperparameters of the model (thresholds for determining TNT-containing pixels) that we tried to train the model on. The decisions on these thresholds were important for determining whether a pixel contained a TNT or not. We needed to optimize between precision (i.e., PPV) and recall (i.e., sensitivity). Once the decision function values were generated on a pixel-wise basis for the images (at the end of Step 2, **Figure 3** in the main manuscript), we classified pixels as TNTs (labeled as 1) or not TNTs (labeled as 0) based on a threshold value, which is one of the hyperparameters of the model. Determining the optimum threshold value required a balance between model precision and recall (specificity), see **Supplementary Figure S3**. Increasing precision would give a rise to FNs whereas increasing the recall would give way to more FPs. We chose the threshold where precision curve crosses the recall curve, which represents an optimal balance between FPs and FNs.
2. There are some cellular extensions that looked like TNTs, but they didn't connect to other cells (so by definition they were not TNTs). Within each subimage (size 512 × 512 pixels), there were cellular extensions in the images that looked like TNTs, but they didn't extend between two cells, which, by definition, disqualified them from being labeled as TNTs. Our model sometimes captured them as TNTs, and this contributed to the increasing number of FPs. Moreover, when forming the training data set, which contained 13,872 subimages per stitched and padded image set, the accepted images for the training dataset were the ones with TNTs located in the middle of the image, as explained above. This created a disadvantage for the model when the test images were fed into the trained model with images where some part of the TNTs were located closer to the edges of the sliding window (i.e., when image sets were partitioned into patches, some of their resulting subimages did not have TNTs located in the middle), see **Supplementary Figure S1**. This clearly increased the number of FNs because one of the cells connected by the TNT was no longer present in the sliding window.
3. TNTs are sometimes mixed with the background color, so the fragments of a single TNT are interpreted by the model as multiple TNTs. This created problems during the automated TNT labeling step. When the model tried to capture a TNT connecting two cells, it correctly predicted some of the TNTs pixel wise, and missed some other pixels due to TNTs pixel intensity being close to that of the background. Thus, the model predicted fragments of a single TNT, and this created problems with the automated TNT labeling step as well as counting the number of TNTs as part of the third step (see **Figure 3** in the main manuscript), because fragments of TNTs were not later determined to constitute a single TNT (i.e., they were all rejected). Recall from section 3.4 of the main manuscript that when counting TNTs, we first formed contours on a morphologically transformed version of the raw images. A morphological transformation helped identify regions of different pixel intensities (ranging from 0 to 255). The contours were defined as curves (i.e., not closed regions) joining contiguous pixels of the same value (either all "1s" or all "0s") along a boundary. Therefore, contours of 1s or 0s were adjacent to each other, and formed boundaries between clusters of pixels with value 1 (i.e., representing TNTs) and clusters of pixels with value 0. After forming these contours, we next matched the stitched image with contours to a version of the same image with the pixels predicted as TNT (that is, "1") or not TNT (that is, "0"). If the number of pixels within a TNT-containing contour (i.e., the prediction of the ML model) after the matching process was between 400 and 2,500 pixels (representing an area range of 44.89 μm^2 to 280.56 μm^2 of the surface of a TNT), we classified and counted that contour as a TNT. During this process, a group of pixels in an image, which correspond to a fragment of a TNTs in the same image, was not counted as one full TNT structure if the number of pixels was less than 400 pixels, even though those pixels may have been correctly predicted to be located within a true TNT. Due to differences in the pixel intensity (range 0-255) between TNTs vs. the background, there were instances in which one full TNT structure was detected by the ML model as multiple fragments that did not meet criteria individually to be defined as TNTs (i.e. less than 400 pixels in area). The precision and recall curves as a function of the threshold of pixel intensity in Model 2 (**Supplementary Figure S3**) was used to determine whether a pixel was contained in a TNT or not.

References

104. Seo, J.H.; Yang, S.; Kang, M.S.; Her, N.G.; Nam, D.H.; Choi, J.H.; Kim, M.H. Automated stitching of microscope images of fluorescence in cells with minimal overlap. *Micron* **2019**, *126*, 102718, doi:10.1016/j.micron.2019.102718.
